# Effectiveness and safety of Liuhedan for treating acute pancreatitis: A protocol for systematic review and meta analysis

**DOI:** 10.1097/MD.0000000000024863

**Published:** 2021-02-26

**Authors:** Tao Cheng, Bo-Fu Liu, Tian-Yong Han, Zhi-Han Gu, Pan Pan, Yu Haifang

**Affiliations:** a Department of Emergency Medicine; b Laboratory of Emergency Medicine, West China Hospital; c Disaster Medical Center, Sichuan University, Chengdu, Sichuan, China.

**Keywords:** acute pancreatitis, duration of hospital stays, liuhedan, meta-analysis, mortality, prognosis, systematic review, traditional Chinese medicine

## Abstract

**Background:**

Liuhedan is a famous traditional Chinese medicine (TCM) formula used to treat acute pancreatitis (AP) in China. However, there is no systematic reviews for the evidence and the therapeutic effectiveness and safety of Liuhedan for treating AP. The aim of this study is to summarize previous evidence, assessing the efficacy and safety of Liuhedan in the treatment of AP.

**Methods:**

We will search the EMBASE, WANFANG DATA, Web of Knowledge, CNKI, PubMed, ClinicalTrials.gov and Cochrane Library from inception to June 30, 2021 to retrieve relevant studies using the search strategy: (“Liuhedan” OR “Liuhe Pill” OR “Liu-He-Dan”) AND (“pancreatitis” OR “pancreatitides”). Two authors independently judged study eligibility and extracted data. Heterogeneity will be examined by computing the *Q* statistic and *I*^2^ statistic.

**Results:**

This study assessed the efficiency and safety of Liuhedan for treating acute pancreatitis.

**Conclusions:**

This study will provide reliable evidence-based evidence for the clinical application of Liuhedan for treating AP.

**Ethics and dissemination:**

Ethical approval is unnecessary as this protocol is only for systematic review and does not involve privacy data. The findings of this study will be disseminated electronically through a peer-review publication or presented at a relevant conference.

## 1 Introduction

Acute Pancreatitis (AP) is a commonly encountered acute abdominal inflammatory disorder^[1]^ and it is part of one of the leading causes of hospitalization among gastrointestinal diseases.^[2]^ The incidence of AP is 34 per 100,000 among human beings, and it is rising worldwide.^[3]^ In the United States, acute pancreatitis leads to 270,000 hospital admissions annually, and inpatient costs exceed 2.5 billion dollars.^[4]^ Despite improvements in critical care, the overall mortality rate is about 5% to 10%, but 36% to 50% in patients with severe pancreatitis.^[1]^ Therefore, it is necessary to improve the managements of patients with AP.

Liuhedan is a famous traditional Chinese medicine (TCM) formula. The medicinal herbs make up this capsule, which contains Radix et Rhizoma Rhei, Cortes Phellodendri, Rhizoma Atractylodis Macrocephal, Radix Angelicae Dahur Cae, Fructus Mume, Herba Menthae, and Mel, etc.^[5,6]^ The basic research on Liuhedan shows that it can inhibit pancreatic secretion and reduce the inflammatory index by anti-inflammatory and immunomodulating, which has the theoretical basis for the treatment of AP. Nowadays, combination of Chinese herb medicine Liuhedan and western medicine has been used to treat AP in China. Despite this formula has been used to treat AP for many years exploring the effectiveness and safety of Liuhedan have been conducted. There are no published systematic reviews and meta-analyses. Therefore, we conducted this systematic review and meta-analysis to clarify the efficacy and safety of Liuhedan for treating AP.

## 2 Methods and analysis

### 2.1 Registration

This protocol of systematic review and meta-analysis is based on the Preferred Reporting Items for Systematic Reviews and meta-analysis Protocols (PRISMA-P) statement guidelines. And the protocol has been registered on International Prospective Register of Systematic Reviews database. The registration number was INPLASY202110050.

### 2.2 Eligibility criteria

The inclusion criteria for the study will include:

1. studies with patient age ≥18 years old, a minimum hospital stay of 24 h and a diagnosis of AP;
2. conference abstracts were only included when they provided adequate relevant information for assessment;
3. the patients with AP were divided into two groups (treated with Liuhedan or without Liuhedan);

Exclusion criteria will include: age < 18 years old, patients with chronic pancreatitis or pancreas carcinoma and patients with incomplete data.

### 2.3 Searching strategy

**Figure 1:**
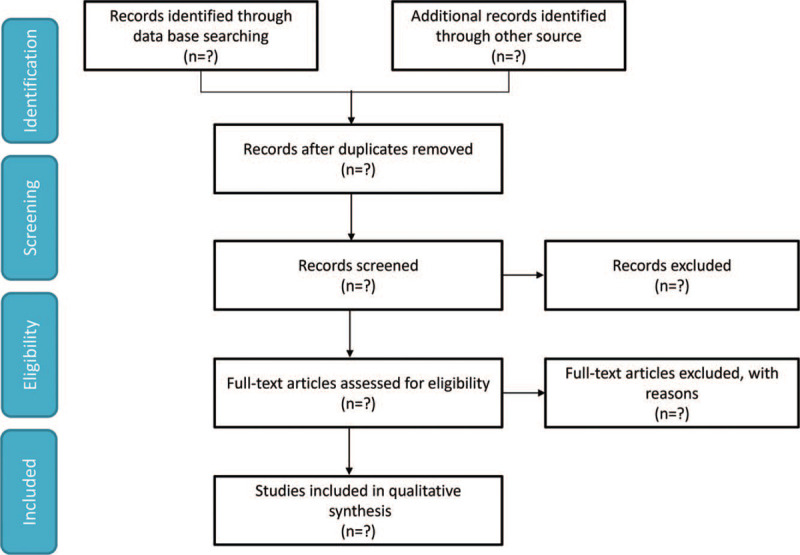
A flow diagram demonstrating the search strategy and study selection process for this study.

We will search the EMBASE, WANFANG DATA, Web of Knowledge, CNKI, PubMed, ClinicalTrials.gov and Cochrane Library from inception to June 30, 2021 to retrieve relevant studies using the search strategy: (“Liuhedan” OR “Liuhe Pill”) AND (“pancreatitis” OR “pancreatitides”). No language restrictions will be applied. We will also search citations of relevant primary and review. Authors of abstract in the meeting will be further searched in PubMed for potential full articles. To minimize the risk of publication bias, we will conduct a comprehensive search that included strategies to find published and unpublished studies. The research summary of the screening flow chart is shown in Figure 1.

### 2.4 Data extraction and risk of bias

Two reviewers will be employed the searching strategy respectively, by reading the papers and scoring them according to the QUADAS-2 checklist^[7]^ and Newcastle–Ottawa Quality Assessment Scale^[8]^; disagreement will be settled by a third opinion. Important information will be abstracted from the included articles in a standardized form by two reviewers. Important information includes the name of the first author, publication year, publication country, type of study, study population, sample size, using of PPIs and outcomes studied (hospital mortality and duration of hospital stays). Risk of bias assessment will be carried out according to the Newcastle–Ottawa Scale (NOS) to rate the internal validity of the individual studies, and funnel plots will be constructed to assess the risk of publication bias.

### 2.5 Statistical analysis

All pairwise meta-analytic calculations will be performed with Review Manager software (RevMan) version 5.3 (Cochrane Collaboration). Heterogeneity will be examined by computing the *Q* statistic and *I*^2^ statistic, and presence of reporting bias by visual inspection of funnel plots. Statistical significance was considered when the *P* value < .05.

## 3 Discussion

Acute pancreatitis is a sudden inflammatory process in the pancreas with variable involvement of nearby organs or other organ systems.^[9–11]^ It is well known that severe AP cases are often associated with severe complications and high mortality.^[12]^ Patients with SAP often complicate systemic inflammatory response syndrome^[13]^ and immune response imbalance.^[14,15]^ Previous researches showed that Liuhedan could inhibit pancreatic secretion and reduce the inflammatory index by anti-inflammatory and immunomodulating and the application of Chinese herbal medicine and electro-acupuncture has led to a marked reduction in morbidity and mortality in China.^[16,17]^

However, the conclusion that Liuhedan improve the prognosis of patients with AP, is controversial and it is not adopted by other country. Therefore, we designed the systematic review and meta-analysis protocol by using the latest data to test the effectiveness and safety of Liuhedan in the treatment of AP. The results of our review will be reported strictly following the PRISMA criteria. And it is hoped that this study could find more rigorous medical evidence for the application of Liuhedan to the treatment of AP, thus providing a reference for clinical practice.

## Acknowledgments

The authors would like to acknowledge the participants and their families for taking part in the study.

## Author contributions

**Conceptualization:** Tao Cheng, Yu Haifang.

**Data curation:** Tao Cheng, Tian-Yong Han, Yu Haifang.

**Formal analysis:** Tao Cheng, Bo-Fu Liu, Tian-Yong Han, Zhi-Han Gu, Pan Pan.

**Funding acquisition:** Yu Haifang.

**Investigation:** Tao Cheng, Tian-Yong Han, Zhi-Han Gu, Pan Pan.

**Methodology:** Tao Cheng, Bo-Fu Liu, Pan Pan.

**Project administration:** Tao Cheng, Bo-Fu Liu, Zhi-Han Gu.

**Resources:** Tao Cheng, Tian-Yong Han, Pan Pan.

**Software:** Tao Cheng, Bo-Fu Liu, Zhi-Han Gu.

**Supervision:** Tao Cheng, Tian-Yong Han, Pan Pan.

**Validation:** Tao Cheng, Bo-Fu Liu, Zhi-Han Gu.

**Visualization:** Tao Cheng, Tian-Yong Han, Yu Haifang.

**Writing – original draft:** Tao Cheng, Bo-Fu Liu, Tian-Yong Han, Zhi-Han Gu, Pan Pan, Yu Haifang.

**Writing – review & editing:** Tao Cheng, Zhi-Han Gu, Yu Haifang.
